# Supplementary figures and images for: Navigating Neighbourhood Opposition and Climate Change: Feasibility and Acceptability of a Play Street Pilot in Sydney, Australia
Source: Int J Environ Res Public Health. 2023 Jan 30;20(3):2476. doi: 10.3390/ijerph20032476 (PMC9916153; doi:10.3390/ijerph20032476)

**Figure S1.** Images of the pilot Play Street, Inner West Sydney, March 2020.

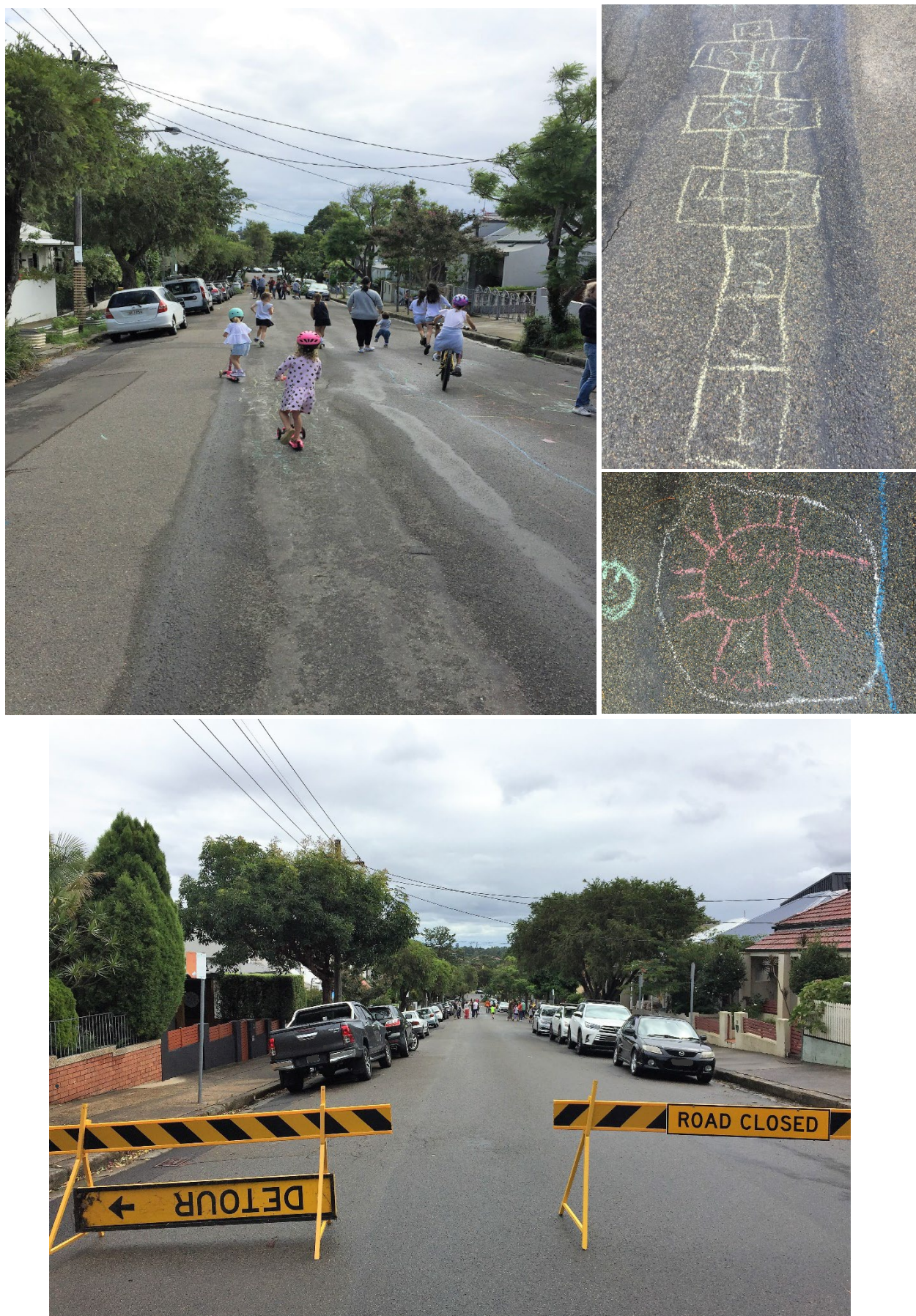

Supplement: Supplementary file 1 [file ijerph-20-02476-s001.zip › ijerph-2065781-supplementary.pdf]
